# Supplementary material for: Reduced diversity and stability of coral-associated bacterial communities and suppressed immune function precedes disease onset in corals
Source: R Soc Open Sci. 2019 Jun 12;6(6):190355. doi: 10.1098/rsos.190355 (PMC6599770; doi:10.1098/rsos.190355)
Supplement: Supplementary figures and tables [file rsos190355supp1.pdf]

Supplementary Material for:

Reduced diversity and stability of coral-associated bacterial communities and suppressed immune function precede disease onset in corals adjacent to reef platforms

F. Joseph Pollock, Joleah B. Lamb, Jeroen A. J. M. van de Water, Hillary A. Smith, Britta Schaffelke, Bette L. Willis, David G. Bourne*

*Corresponding Author: David G. Bourne, James Cook University, Australian Institute of Marine Science, and AIMS@JCU (david.bourne@jcu.edu.au)

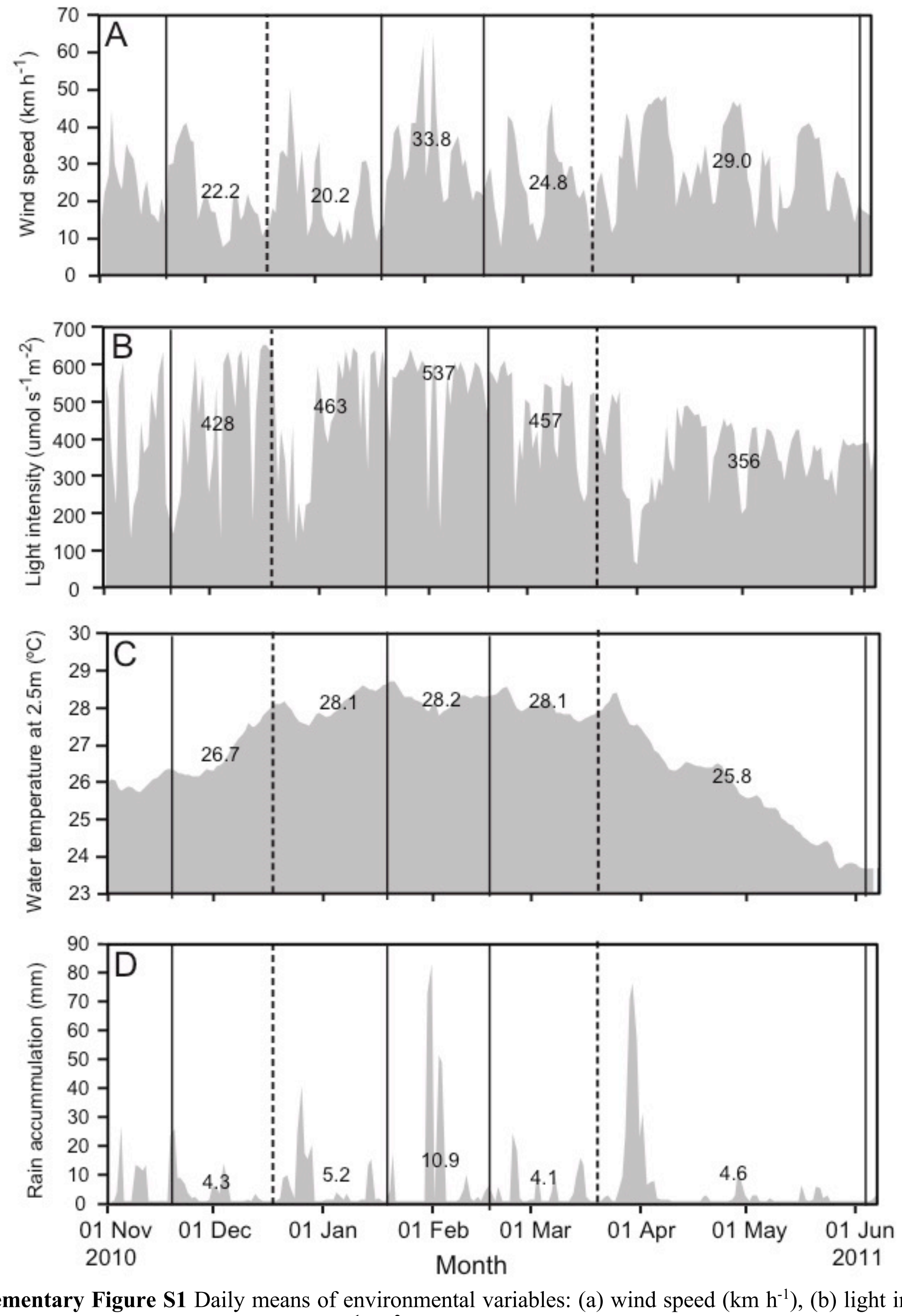

Supplementary Figure S1 Daily means of environmental variables: (a) wind speed (km h^{-1}), (b) light intensity (photosynthetically active radiation, µmol s^{-1} m^{-2}), (c) water temperature (2.5 m depth, ºC), and (d) rainfall accumulation (mm), adapted from van de Water *et al.* 2015. Bold dashed vertical lines indicate sampling time points for water quality, bacterial community and immune function profiling. Non-bold vertical dashed lines indicate time points for visual colony health assessments only. Numbers between vertical lines represent mean daily values between time points. Environmental data obtained from the Australian Institute of Marine Science (AIMS) monitoring station located on the main tourist platform at Hardy Reef, figure adapted from van de Water *et al.* 2015.

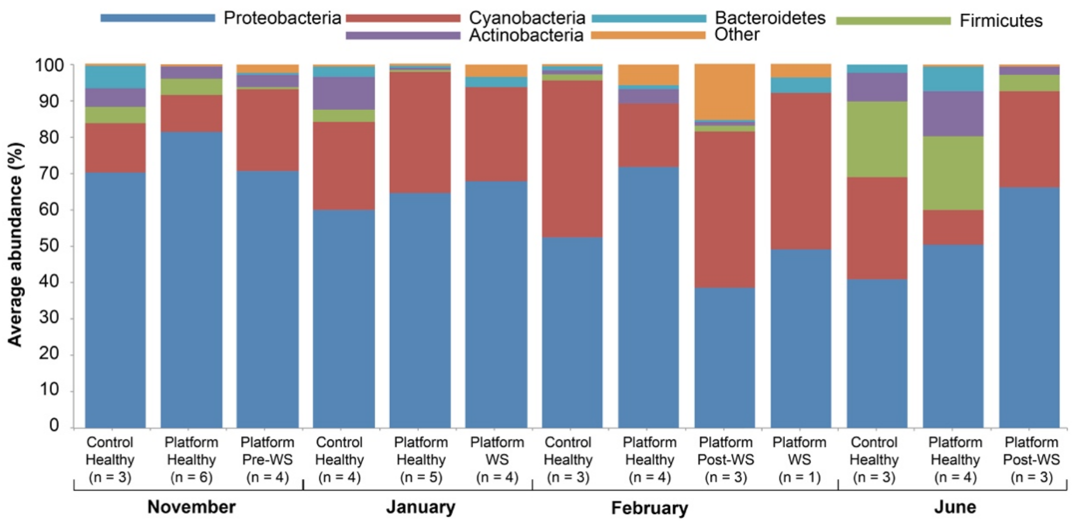

Supplementary Figure S2 Average abundance of five major taxonomic divisions (all taxa with < 1% average abundance are combined in "Other") comprising bacterial communities associated with *A. millepora* across two locations (control and platform) and four health states (healthy, pre-WS, WS and post-WS).

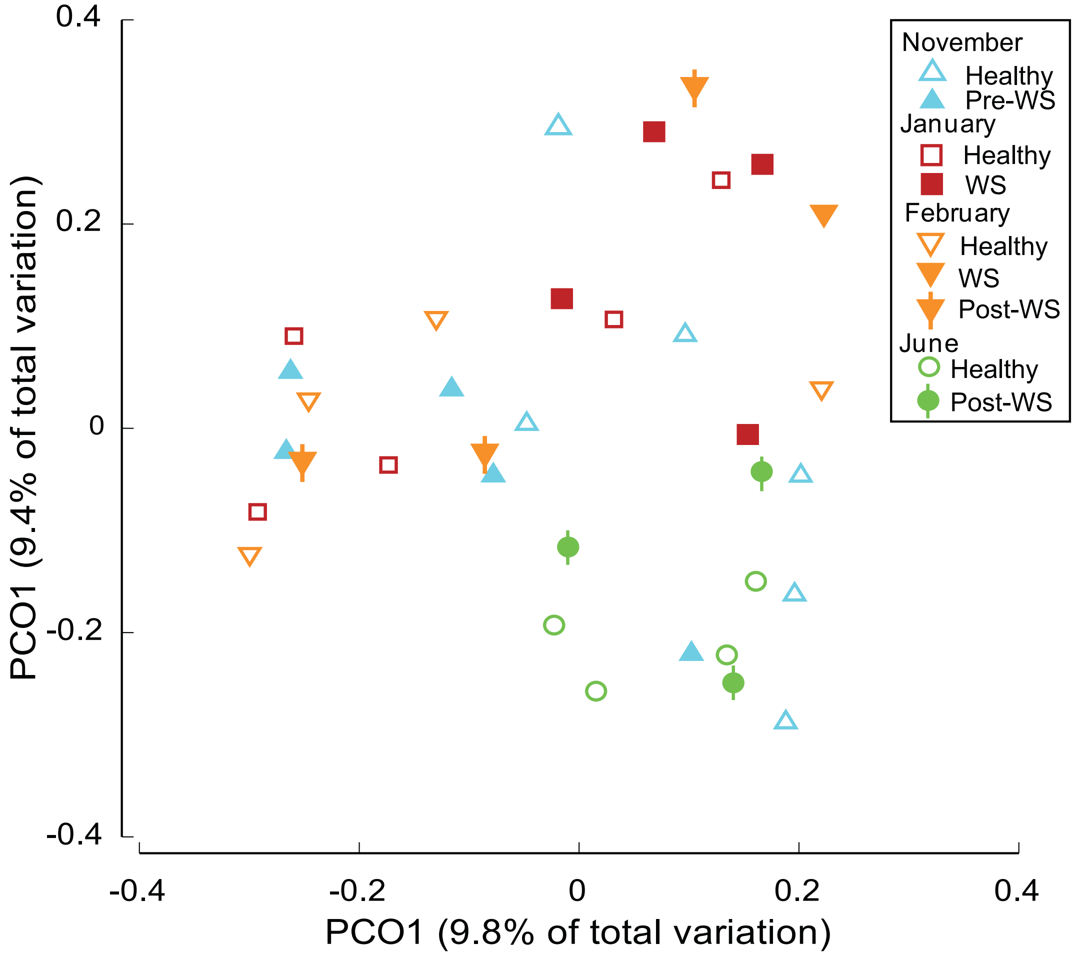

Supplementary Figure S3 Two-dimensional principal coordinates plot visualizing separation between bacterial communities (unweighted UniFrac distance) associated with colonies of *A. millepora* located at platform sites that remained visually healthy throughout the study (white symbols), colonies prior to visual signs of white syndrome (pre-WS, grey symbols), colonies displaying signs of WS in January and February (black symbols) and colonies without WS lesion progression (post-WS, black hash symbols). November = blue triangles, January = red squares; February = orange triangles; June = green circles.

Supplementary Table S1 Results of a two-way crossed permutational analysis of variance (PERMANOVA) of water quality parameters. Water quality parameters measured include concentrations of dissolved inorganic nutrients (DIP, DIN, silicate), dissolved organic nutrients (DON, DOP, DOC) and salinity.

Source	df	SS	MS	Pseudo-F	P(perm)	Unique perms
**Month**	**2**	**6625.4**	**3312.7**	**18.96**	**0.0001**	**9932**
Location	1	128.8	128.8	0.74	0.6069	9960
Month x Location	2	577.6	288.8	1.65	0.1152	9943
Res	39	6826.3	175.0			
Total	44	14965				

Supplementary Table S2 Water quality concentrations [mean (stdev)] at each site (n = 5 replicates at each site per month) and physical parameters [mean (stdev)] calculated using daily values from 14 days prior to and including the sampling date for each month. DIP = dissolved inorganic phosphorus, Si = silicate, DIN = dissolved inorganic nitrogen, DOP = dissolved organic phosphorus, DON = dissolved organic nitrogen, DOC = dissolved organic carbon.

	**Tourist platform**			**Unused platform**			**Control**		
Variable	January	February	June	January	February	June	January	February	June
DIP (µm/L)	0.07 (0.005)	0.05 (0.008)	0.07 (0.017)	0.07 (0.005)	0.07 (0.007)	0.07 (0.015)	0.06 (0.005)	0.07 (0.014)	0.07 (0.006)
Si (mg/L)	1.72 (0.06)	2.15 (0.049)	1.09 (0.047)	1.99 (0.124)	1.61 (0.096)	1.14 (0.055)	2 (0.156)	1.36 (0.06)	1.31 (0.024)
DOC (µm/L)	1.02 (0.11)	0.7 (0.026)	0.81 (0.019)	0.82 (0.019)	0.87 (0.049)	0.82 (0.016)	1 (0.083)	0.8 (0.011)	0.87 (0.015)
Salinity (ppt)	34.7 (0.002)	34.78 (0.003)	35.51 (0.102)	34.69 (0.004)	34.79 (0)	35.31 (0.02)	34.68 (0.007)	34.8 (0.005)	35.34 (0.044)
DIN (µm/L)	0.45 (0.07)	0.889 (0.175)	0.363 (0.113)	0.974 (0.377)	0.779 (0.13)	0.433 (0.05)	0.707 (0.234)	0.867 (0.039)	0.468 (0.053)
DON (µm/L)	9.25 (5.09)	5.57 (0.655)	5.203 (0.772)	8.56 (3.932)	5.214 (0.568)	7.46 (5.284)	9.486 (3.566)	5.159 (0.47)	6.83 (1.351)
DOP (µm/L)	0.14 (0.11)	0.205 (0.022)	0.221 (0.07)	0.308 (0.181)	0.119 (0.06)	0.182 (0.084)	0.13 (0.069)	0.117 (0.044)	0.231 (0.057)
Water temperature (ºC)	28.1 (0.3)	28.3 (0.2)	25.9 (1.4)						
Rain accumulation (mm)	5.1 (8.9)	10.9 (23.2)	4.6 (13.5)						
Light intensity (µmol s^1m^{-2})	470.6 (168.4)	535.1 (122.2)	358.8 (95.0)						

Supplementary Table S3 Results of the SIMPER analyses showing species responsible for the 1% or greater differences in bacterial communities associated with apparently healthy colonies of *A. millepora* between platform and control sites in January.

Operational taxonomic unit (OTU)					Samples with OTU read (%)		Contribution to separation (%)	
Phylum	Class	Order	Family	Genus	Platform	Control	Individual	Cumulative
Firmicutes	Clostridia	Clostridiales	Peptostreptococcaceae	Tepidibacter	0	100	1.9	1.9
Proteobacteria	Alphaproteobacteria	Sphingomonadales	Sphingomonadaceae	-	0	100	1.9	3.8
Proteobacteria	Betaproteobacteria	BVC71	-	-	0	100	1.9	5.7
Proteobacteria	Betaproteobacteria	Burkholderiales	Oxalobacteraceae	Janthinobacterium	0	100	1.9	7.6
Cyanobacteria	Synechococcophycideae	Synechococcales	Synechococcaceae	Prochlorococcus	20	100	1.5	9.1
Actinobacteria	Actinobacteria	Actinomycetales	Nocardioidaceae	-	0	75	1.5	10.5
Firmicutes	Bacilli	Bacillales	Bacillaceae	Geobacillus	0	75	1.3	11.9
Proteobacteria	Gammaproteobacteria	Pseudomonadales	Pseudomonadaceae	Pseudomonas	0	75	1.3	13.2
Proteobacteria	Alphaproteobacteria	Rhodospirillales	Rhodospirillaceae	-	20	75	1.3	14.5
Proteobacteria	Alphaproteobacteria	Rhizobiales	-	-	20	75	1.3	15.8
Proteobacteria	Betaproteobacteria	Burkholderiales	Burkholderiaceae	Burkholderia	80	25	1.3	17.0
Proteobacteria	Betaproteobacteria	Burkholderiales	Oxalobacteraceae	-	20	75	1.3	18.3
Proteobacteria	Gammaproteobacteria	Enterobacteriales	Enterobacteriaceae	Gluconacetobacter	20	75	1.3	19.6
Actinobacteria	Actinobacteria	Actinomycetales	Corynebacteriaceae	Corynebacterium	20	75	1.2	20.7
Proteobacteria	Alphaproteobacteria	Sphingomonadales	Sphingomonadaceae	Sphingomonas	20	75	1.2	21.9
Proteobacteria	Gammaproteobacteria	Pseudomonadales	Moraxellaceae	Acinetobacter	20	75	1.2	23.1
Actinobacteria	Actinobacteria	Actinomycetales	Propionibacteriaceae	Propionibacterium	40	100	1.1	24.2
Cyanobacteria	Chloroplast	CAB-I	-	-	40	100	1.1	25.2
Proteobacteria	Alphaproteobacteria	Rickettsiales	Pelagibacteraceae	-	0	50	1.1	26.3
Proteobacteria	Betaproteobacteria	Burkholderiales	Comamonadaceae	Roseateles	0	50	1.1	27.3
Proteobacteria	Gammaproteobacteria	Alteromonadales	Colwelliaceae	Thalassomonas	0	50	1.1	28.4
Actinobacteria	Actinobacteria	Actinomycetales	-	-	0	50	1.0	29.4
Proteobacteria	Alphaproteobacteria	Rhodospirillales	Acetobacteraceae	Roseomonas	0	50	1.0	30.4
Proteobacteria	Alphaproteobacteria	Rickettsiales	-	-	0	50	1.0	31.4

Supplementary Table S4 Results of permutational analyses of variance (PERMANOVA) of OTU-level bacterial community composition for a) a two-factor analysis of Month and Location effects over the course of the 8-month study, b-e) one-way assessments of Location effect during four sampling months (November, January, February and June), and f) a two-factor analysis of Location and Health State effects over the course of the 8-month study.

Source	df	SS	MS	Pseudo-F	P(perm)	Unique perms
a) Location x Month (all months)						
Month	3	0.89173	0.29724	1.0405	0.3418	9719
**Location**	**1**	**0.40835**	**0.40835**	**1.4295**	**0.0331**	**9844**
Month x Location	3	0.98897	0.32966	1.154	0.099	9761
Res	24	6.8559	0.28566			
Total	31	9.1555				
b) Location (November)						
Location	1	0.30503	0.30503	1.1374	0.2117	164
Res	9	2.4136	0.26817			
Total	10	2.7186				
c) Location (January)						
**Location**	**1**	**0.49347**	**0.49347**	**1.5795**	**0.0049**	**210**
Res	8	2.4993	0.31241			
Total	9	2.9928				
d) Location (February)						
Location	1	0.27947	0.27947	0.96128	0.5022	56
Res	6	1.7443	0.29072			
Total	7	2.0238				
e) Location (June)						
**Location**	**1**	**0.3605**	**0.3605**	**1.3195**	**0.03**	**35**
Res	5	1.3661	0.27321			
Total	6	1.7266				
f) Location x Health State (all months)						
Month	3	1.0204	0.34013	1.1285	0.1357	9759
Health State	1	0.35015	0.35015	1.1618	0.1908	9834
Month x Health State	3	0.83955	0.27985	0.92853	0.74	9730
Res	26	7.8362	0.30139			
Total	33	10.042				

Supplementary Table S5 Results of the SIMPER analyses showing species responsible for 1% or more of the differences in bacterial communities associated with apparently healthy colonies of *A. millepora* between platform and control sites in June.

Operational taxonomic unit (OTU)					Samples with OTU read (%)		Contribution to separation (%)	
Phylum	Class	Order	Family	Genus	Pontoon	Control	Individual	Cumulative
Actinobacteria	Actinobacteria	Actinomycetales	Micrococcaceae	Micrococcus	100	0	2.7	2.7
Proteobacteria	Betaproteobacteria	BVC71	-	-	100	0	2.7	5.4
Proteobacteria	Betaproteobacteria	Burkholderiales	Comamonadaceae	Variovorax	100	0	2.7	8.1
Proteobacteria	Betaproteobacteria	Burkholderiales	Oxalobacteraceae	Janthinobacterium	100	0	2.7	10.8
Cyanobacteria	Chloroplast	CAB-I	-	-	100	33	2.0	12.8
Cyanobacteria	Chloroplast	Chlorophyta	Ulvophyceae	-	100	33	2.0	14.8
Firmicutes	Clostridia	Clostridiales	Peptostreptococcaceae	Tepidibacter	100	33	2.0	16.7
Proteobacteria	Alphaproteobacteria	Sphingomonadales	Sphingomonadaceae	-	100	33	1.9	18.7
Proteobacteria	Betaproteobacteria	Burkholderiales	Oxalobacteraceae	-	100	33	1.9	20.6
Actinobacteria	Actinobacteria	Actinomycetales	Microbacteriaceae	Microbacterium	75	0	1.8	22.4
Actinobacteria	Actinobacteria	Actinomycetales	Corynebacteriaceae	Corynebacterium	75	33	1.7	24.1
Proteobacteria	Alphaproteobacteria	Sphingomonadales	Sphingomonadaceae	Novosphingobium	50	0	1.6	25.7
Proteobacteria	Alphaproteobacteria	Rhodospirillales	Acetobacteraceae	Roseomonas	50	0	1.5	27.1
Proteobacteria	Deltaproteobacteria	-	-	-	50	33	1.4	28.5
Firmicutes	Bacilli	Lactobacillales	Streptococcaceae	-	25	67	1.4	30.0
Firmicutes	Bacilli	Lactobacillales	Streptococcaceae	Streptococcus	25	67	1.4	31.4
Proteobacteria	Gammaproteobacteria	Xanthomonadales	Xanthomonadaceae	Stenotrophomonas	25	67	1.4	32.8
Actinobacteria	Actinobacteria	Actinomycetales	Micrococcaceae	Arthrobacter	50	0	1.4	34.2
Firmicutes	Clostridia	Clostridiales	-	-	50	33	1.4	35.6
Cyanobacteria	Synechococcophycideae	Synechococcales	Synechococcaceae	Prochlorococcus	50	67	1.3	36.9
Proteobacteria	Betaproteobacteria	Burkholderiales	Comamonadaceae	Tepidimonas	50	0	1.3	38.2
Proteobacteria	Gammaproteobacteria	Pseudomonadales	Pseudomonadaceae	-	50	33	1.3	39.5
Proteobacteria	Gammaproteobacteria	Enterobacteriales	Enterobacteriaceae	Gluconacetobacter	50	33	1.3	40.8
Proteobacteria	Gammaproteobacteria	Pseudomonadales	Pseudomonadaceae	Pseudomonas	75	67	1.3	42.0
Bacteroidetes	Flavobacteriia	Flavobacteriales	Flavobacteriaceae	-	50	33	1.2	43.3
Proteobacteria	Alphaproteobacteria	Rhizobiales	-	-	50	0	1.2	44.5
Proteobacteria	Alphaproteobacteria	Sphingomonadales	Erythrobacteraceae	-	50	0	1.2	45.7
Proteobacteria	Alphaproteobacteria	Rhodospirillales	Rhodospirillaceae	Skermanella	0	33	1.2	46.9
Proteobacteria	Betaproteobacteria	Burkholderiales	Oxalobacteraceae	Ralstonia	0	33	1.2	48.1
Proteobacteria	Gammaproteobacteria	Oceanospirillales	Endozoicimonaceae	-	100	67	1.2	49.3
Proteobacteria	Gammaproteobacteria	Pseudomonadales	Moraxellaceae	Acinetobacter	100	67	1.2	50.5
Firmicutes	Bacilli	Bacillales	Staphylococcaceae	Staphylococcus	25	33	1.2	51.7
Actinobacteria	Actinobacteria	Actinomycetales	Microbacteriaceae	-	50	0	1.2	52.8
Cyanobacteria	Chloroplast	Streptophyta	-	-	50	0	1.2	54.0
Firmicutes	Bacilli	Bacillales	Alicyclobacillaceae	Alicyclobacillus	25	33	1.0	55.0
Actinobacteria	Actinobacteria	Actinomycetales	-	-	25	33	1.0	56.0
Proteobacteria	Alphaproteobacteria	Sphingomonadales	Sphingomonadaceae	Sphingomonas	25	33	1.0	57.0
